# Wahlfach Robotische Chirurgie – Faszination Lehre(n)?

**DOI:** 10.1007/s00120-021-01756-6

**Published:** 2022-01-17

**Authors:** Philip Zeuschner, Philippe Becker, Julia Heinzelbecker, Johannes Linxweiler, Stefan Siemer, Michael Stöckle, Matthias Saar

**Affiliations:** Klinik für Urologie und Kinderurologie, Universitätsklinikum des Saarlandes, Kirrberger Straße 100, 66421 Homburg/Saar, Deutschland

**Keywords:** Robotische Chirurgie, Robotik, Simulationstraining, Medizinische Lehre, Medizinstudium, Robotic surgical procedures, Robotics, Simulation training, Medical education, Medical studies

## Abstract

**Hintergrund:**

Auch wenn sich roboterassistiertes Operieren zu einem verbreiteten Standardverfahren in einigen chirurgischen Fächern entwickelt hat, ist es im Lehrplan heutiger Medizinstudierender unterrepräsentiert.

**Fragestellung:**

Wir berichten vom deutschlandweit ersten Wahlfach „Robotische Chirurgie“ für Studierende an einer urologischen Universitätsklinik.

**Material und Methoden:**

In einer Kleingruppe mit zehn Studierenden wurden in sechs Treffen à 2 h theoretische Grundlagen und praktische Fertigkeiten in der robotischen Chirurgie vermittelt, inklusive einer Hospitation während einer urologischen roboterassistierten Operation. Der Zuwachs an Wissen (10 MCQ-Fragen) und Fähigkeiten (Übungen Camera 0, Clutch, Sea Spikes 1) an einem robotischen Simulationssystem wurde quantifiziert und die studentische Einschätzung evaluiert.

**Ergebnisse:**

Bei den 10 Teilnehmenden war ein signifikanter Wissenszuwachs messbar, am Ende wurden in derselben theoretischen Prüfung im Median 3,5 mehr korrekte Antworten gegeben (*p* = 0,011). In zwei von drei praktischen Übungen stieg die Gesamtpunktzahl signifikant an (Camera 0 und Sea Spikes 1, für beide *p* < 0,05), in der Übung „Clutch“ verbesserte sich nur die Bewegungsökonomie (*p* = 0,028). Das Modul wurde (sehr) gut bewertet und die Teilnehmenden konnten sich am Ende deutlich stärker vorstellen, später Urologe/in zu werden (*p* = 0,007).

**Schlussfolgerungen:**

Bei einem Bedarf von studentischer Seite, mehr über roboterassistierte Operationen zu lernen, erscheint ein Wahlfach als geeignetes Format, um theoretische Grundlagen und praktische Fertigkeiten in der robotischen (urologischen) Chirurgie zu vermitteln. Zusätzlich hat es das Potenzial, auf das Fachgebiet Urologie aufmerksam zu machen und könnte potenziell neue Kolleginnen und Kollegen gewinnen.

**Zusatzmaterial online:**

Die Online-Version dieses Beitrags (10.1007/s00120-021-01756-6) enthält als zusätzliches Material das in diesem Beitrag durchgeführte Eingangstestat zur Prüfung des Vorwissens der Studierenden in einer theoretischen Prüfung (10 Multiple-choice-Fragen).

## Abstract (en)

**Background:**

Even though robot-assisted operations have evolved to a standard procedure in surgery, they are underrepresented in the curriculum of current medical students.

**Objectives:**

We present our experience and findings in Germany’s first elective “Robot-assisted surgery” at a urological department for undergraduate medical students.

**Materials and methods:**

Ten undergraduates in their final years were taught the theoretical basics and practical skills in robot-assisted surgery within six lessons each lasting 2 h, including the opportunity to observe a live robot-assisted surgery. The increase of knowledge (ten multiple-choice questions) and skills (exercises Camera 0, Clutch, and Sea Spikes 1) on a robotic simulation device were quantified including an evaluation of the student’s perspective.

**Results:**

The 10 participants had a significant increase in knowledge and gave at a median of 3.5 additional correct answers in the final assessment (*p* = 0.011). For two out of three practical exercises, the overall score significantly increased (Camera 0 and Sea Spikes 1, for both *p* < 0.05), but for the exercise “Clutch”, only economy of motion significantly improved (*p* = 0.028). The elective was evaluated (very) good and the willingness of the participants to become urologists significantly increased (*p* = 0.007).

**Conclusion:**

There is a great interest of many undergraduate medical students in robot-assisted surgery. Offering an elective appears to be an excellent format to teach the theoretical background and practical skills in robotic (urologic) surgery. Moreover, such an elective could raise more attention to the field of urology and might attract future colleagues.

## Einleitung

Seit der Food and Drug (FDA)-Zulassung des DaVinci®-Operationssystems im Jahr 2000 hat sich die robotische Chirurgie als Standardverfahren in der Urologie und in vielen weiteren operativen Fächern etabliert. Auch wenn eine signifikante Zahl (mittel)großer Kliniken heute roboterassistierte Operationen anbietet, spielt die robotische Chirurgie im Lehrplan von Medizinstudenten nur eine untergeordnete Rolle.

Demgegenüber existieren bereits diverse strukturierte Curricula und Trainingsprogramme für angehende robotische Operateure, zu denen u. a. das 6‑monatige Deutsche Robotercurriculum der Deutschen Gesellschaft für Robotische Urologie (DRGU) oder als europäisches Pendant das ERUS (European Association of Urology Robotic Urology Section) Robotic Curriculum zählen [3]. Diesen Programmen liegt die Erkenntnis zugrunde, dass ein strukturiertes Training insbesondere unter Einbeziehung von Simulatortraining in kürzerer Zeit einen größeren Lernerfolg mit besseren operativen Ergebnissen ermöglicht [14]. Trainingsprogramme für den in der Regel unerlässlichen Bedside-Assistenten sind schon deutlich seltener, auch wenn gezeigt werden konnte, dass dessen Erfahrung bei komplexen robotischen Operateuren ebenso einen signifikanten Einfluss auf das operative Ergebnis hat [2, 23]. Prinzipiell könnte es noch sinnvoller sein, mit einer strukturierten Ausbildung in der robotischen Chirurgie bereits im Medizinstudium zu beginnen. Einerseits sind robotische Operationen aus dem klinischen Alltag in vielen urologischen Kliniken nicht mehr wegzudenken und sollten deswegen Bestandteil der urologischen Lehre sein. Andererseits könnte ein solches Programm auch dazu dienen, aus berufspolitischer Sicht vermehrtes Interesse an der Urologie zu erzeugen und talentierte zukünftige Kolleginnen und Kollegen frühzeitig zu gewinnen [16, 21].

Unsere Klinik verfügt über zwei DaVinci®-Operationssysteme vom Typ „X“ und einen DaVinci® SimNow ®-Simulator (Intuitive Surgical, Sunnyvale, CA, USA), der ein virtuelles Üben und Operieren an der Operationskonsole ermöglicht. Um Erfahrungen in der Implementierung der robotischen Chirurgie in die urologische Lehre zu sammeln, haben wir kürzlich erstmalig für eine urologische Universitätsklinik in Deutschland das Wahlfach „Robotische Chirurgie“ in einer Kleingruppe von 10 Studierenden aus den klinischen Semestern organisiert. In 6 Treffen à 2 h wurden theoretische Grundlagen und praktische Fertigkeiten in der robotischen Chirurgie vermittelt inklusive einer Hospitation während einer roboterassistierten Operation. Der Zuwachs an Wissen und Fertigkeiten wurde quantifiziert und die studentische Einschätzung evaluiert.

## Methodik

Nach Verzögerungen aufgrund der Coronapandemie konnte der erste Termin unter strenger Einhaltung der coronabedingten Vorsichtsmaßnahmen (Abstandsregeln, Tragen eines medizinischen Mund-Nase-Schutz, Nachweis einer abgeschlossenen Impfung, durchgemachten Infektion oder tagesaktueller negativer Schnelltest) auf Mitte Juni 2021 festgesetzt wurde. Alle Treffen fanden im leeren robotischen Operationssaal der Klinik für Urologie und Kinderurologie statt.

**Figure 1:**
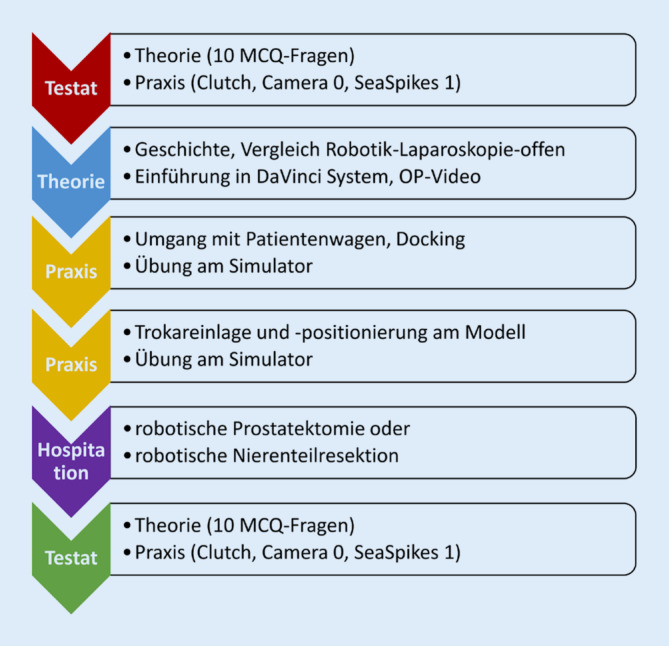

Der Ablauf des Wahlfachs ist in Abb. 1 dargestellt. Während des Eingangstestats am ersten Treffen wurde das Vorwissen der Studierenden in einer theoretischen Prüfung (10 Multiple-choice-Fragen, s. Infobox 1) erhoben. Zudem erfolgte die Messung der praktischen Fähigkeiten mit drei verschiedenen Übungen an der Konsole eines DaVinci®-X-Systems mit angedocktem SimNow®-Simulator, bewusst mit nur sehr kurzer vorheriger Einführung (Clutch: Verwendung der Kupplung, Camera 0: Steuerung der Kamera, Sea Spikes 1: Bedienung der Greifarme). Im zweiten Treffen erfolgte online die Vermittlung der theoretischen Grundlagen zur Historie robotischer Chirurgie, eine ausführliche Einführung in das robotische Operationssystem inklusive verschiedener Docking-Strategien und ein kritischer Vergleich von robotischem, laparoskopischen und offenen Operieren. Zudem wurden Besonderheiten der Robotik, wie die Notwendigkeit einer guten Kommunikation zwischen Bedside-Assistent und Operateur besprochen und gemeinsam eine vorher aufgezeichnete robotische radikale Prostatektomie analysiert. An Treffen 3 und 4 lag der Schwerpunkt auf einem möglichst großen Praxisanteil mit Einweisung in den Umgang mit dem Patientenwagen, Feinheiten der Trokareinlage und weiteren Übungen am Modell sowie Simulator. Eine Hospitation als 5. Bestandteil des Moduls wurde individuell in Zweiergruppen vereinbart. Während des letzten 6.Treffens wurden zur Projekteffektemessung dieselbe theoretische und praktische Prüfung wiederholt und eine anonyme Evaluation durchgeführt. Eine individuelle Bewertung der Teilnehmenden entfiel, weil es sich um ein rein freiwilliges Wahlfach handelte, für das keine Benotung vorgesehen ist.

Die statistische Auswertung erfolgte mit SPSS (IBM, Armonk, USA). Häufigkeiten wurden als Median (Range) angegeben, die verbundenen Stichproben mit dem Wilcoxon-Test verglichen, *p* < 0,05 galt als statistisch signifikant. Die Durchführung des Wahlfachs erfolgte im Rahmen des Teach-the-teacher-Kurses des Universitätsklinikums des Saarlandes und wurde durch den Studiendekan genehmigt.

## Ergebnisse

Bei mehr als 30 Anfragen wurde das Wahlfach mit 6 Studentinnen und 4 Studenten aus dem 6. bis 10. Semester begonnen, die Teilnehmerauswahl erfolgte nach dem Prinzip „first come, first serve“. Die durchschnittliche Teilnahmequote betrug 83 %, wobei bisher noch nicht alle individuellen Hospitationen stattgefunden haben. Ein Teilnehmender hatte zuvor ein Pflegepraktikum in der Urologie absolviert und 2 weitere eine Famulatur in einer urologischen Klinik.

Die praktischen Treffen bedurften einer guten Planung und Abstimmung innerhalb der Abteilung wie interdisziplinär mit der Anästhesie, weil sichergestellt werden musste, dass ein bestimmter OP-Saal von 17 bis 19 Uhr trotz etwaiger Notoperationen frei sein musste. Um die Wartezeiten zu verkürzen, bis die Teilnehmenden wieder am Simulator üben können, wurde die Gruppe ab dem 3. Treffen zweigeteilt und dann in noch kleineren Teams mit 5 Teilnehmenden je 1 h unterrichtet.

**Figure 2:**
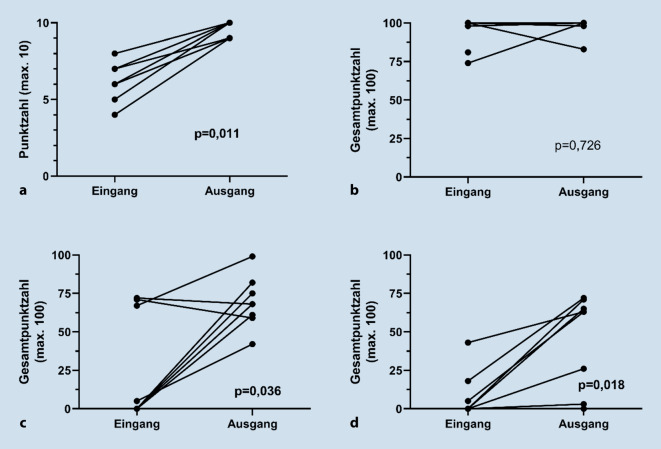

Im theoretischen Ein- und Ausgangstestat ließ sich ein signifikanter Wissenszuwachs aller Teilnehmender feststellen. Bei anfänglich im Median 6 (Range 4–8) richtigen Antworten wurden im Ausgangstestat 9,5 (9–10) und damit signifikant mehr Fragen korrekt beantwortet (*p* = 0,011, s. Abb. 2). Im Praxisteil wurden in den Übungen „Camera 0“ und „Sea Spikes 1“ am Ende ebenfalls signifikant höhere Punktwerte erzielt (*p* = 0,036 und *p* = 0,018). Bei der Übung „Clutch“ ließ sich keine signifikante Veränderung in der Gesamtpunktzahl feststellen. Bereits im Eingangstestat wurden hier im Median 98 (Range 74–100) von 100 möglichen Punkten erreicht, am Ende des Wahlfachs 100 (83–100) Punkte. Trotzdem lag auch bei dieser Übung eine signifikant verbesserte Bewegungsökonomie vor, da die mediane Bewegungsstrecke von 214,8 cm (Range 166,1–379,5) auf 166,9 cm (160,6–212,5) abnahm (*p* = 0,028).

Acht von 10 Studierenden nahmen an der Evaluation teil und bewerteten das Modul als (sehr) gut (s. Tab. 1). Im Freitext wurde als besonders positiv hervorgehoben, dass sich in einer Kleingruppe mit hohem Praxisanteil viel Zeit für Fragen genommen wurde. Es wurde vorgeschlagen, noch einen zweiten Teil für „Fortgeschrittene“ anzubieten und die einzelnen Treffen länger zu gestalten, damit noch mehr Übungszeit an der Konsole besteht.

**Table 1:** 

	1	2	3	4	5	6	x_Med_
Wie bewerten Sie das Eingangstestat?	7	–	–	–	–	–	1,0
Wie bewerten Sie den Theorieteil?	3	4	–	–	–	–	1,5
Wie bewerten Sie den dritten Praxisteil?	7	–	–	–	–	–	1,0
Wie bewerten Sie den vierten Praxisteil?	6	1	–	–	–	–	1,14
Wie bewerten Sie die Hospitation?	4	–	–	–	–	–	1,0
Wie bewerten Sie das Ausgangstestat?	7	–	–	–	–	–	1,0
Wie bewerten Sie das Wahlfach *insgesamt*?	8	–	–	–	–	–	1,0
Wie bewerten Sie die Organisation des Wahlfachs?	8	–	–	–	–	–	1,0
Wie bewerten Sie den didaktischen Aufbau?	8	–	–	–	–	–	1,0
Wie bewerten Sie die Motivation des Dozenten?	8	–	–	–	–	–	1,0

Zum Zeitpunkt des Wahlfachs befand sich noch kein Teilnehmender im praktischen Jahr (PJ), einer hatte schon vorher beabsichtigt, dies in der Urologie abzuleisten. Nach Abschluss des Wahlfachs haben 4 Studierende ihr PJ fest ohne Urologie geplant, wobei zumindest ein Teilnehmender dies möglicherweise noch ändern möchte. Bei 4 weiteren Studierenden zählt Urologie mit zur engeren Auswahl.

**Figure 3:**
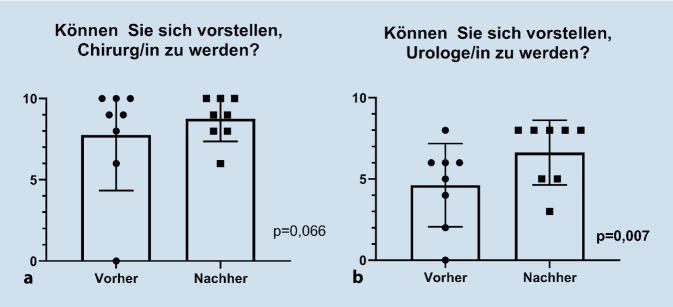

Am Ende des Wahlfachs konnten sich die Studierenden deutlich besser vorstellen, später als Urologe/in tätig zu werden (*p* = 0,007, s. Abb. 3). Auch wenn die Bereitschaft, später als Chirurg/in zu arbeiten, ebenfalls zunahm, war dieser Unterschied nicht statistisch signifikant (*p* = 0,066).

## Diskussion

Roboterassistierte Operationen sind heute nicht mehr aus dem Alltag vieler urologischer Kliniken wegzudenken. Im Jahr 2018 wurden in Deutschland von insgesamt 11.492 radikalen Prostatektomien 39,1 % robotisch und damit fast doppelt so viele wie offen (2253, entsprechend 19,6 %) durchgeführt. Der Anteil laparoskopischer radikaler Prostatektomien war mit 41,3 % nur knapp höher [7]. Bei den Nierenteilresektionen im Jahr 2019 in Deutschland wurden demgegenüber mehr als doppelt so viele Eingriffe robotisch wie laparoskopisch durchgeführt (28,8 % vs. 13 %), und dieser Anteil stieg im Jahr 2020 auf 36,9 % (3577 von 9700 Eingriffen; [24]). Demzufolge sollte die Robotik auch ein fester Bestandteil der (urologischen) Lehre im Medizinstudium sein. Das Medizinstudium befindet sich derzeit mit dem „Masterplan Medizinstudium 2020“ in einem Umbruch und der nationale kompetenzbasierte Lernzielkatalog (NKLM) wurde kürzlich überarbeitet [4, 5]. Dadurch ergeben sich einerseits Chancen für die Urologie, da ein stärkerer Fokus auf die Anwendungsebene im Medizinstudium gelegt wird und viele Prüfungen bspw. im OSCE-Format („objective structured clinical examination“) abgehalten werden sollen [18]. Andererseits wird auch die Digitalisierung in der Medizin einen weiteren Kernaspekt im Medizinstudium spielen, die roboterassistierte Chirurgie ist jedoch weiterhin unterrepräsentiert [22]. Um die urologische Lehre in unserer Klinik um Lehrinhalte der robotischen Chirurgie zu erweitern, haben wir kürzlich deutschlandweit erstmalig in einer urologischen Klinik das Wahlfach „Robotische Chirurgie“ angeboten. In einer Kleingruppe von 10 Studierenden der klinischen Semester wurden in 6 Treffen à 2 h theoretische Inhalte und praktische Fertigkeiten vermittelt. Die Teilnehmenden hatten nicht nur einen signifikanten Zuwachs an Wissen und Fertigkeiten, auch ihre Bereitschaft, später in der Urologie tätig zu werden, stieg messbar an.

Bislang existieren nur wenige strukturierte und evaluierte Lehrkonzepte der robotischen Chirurgie für Studierende. Das 2‑wöchige Curriculum von Mullens et al. ähnelt einem Intensivkurs mit sehr hohem Praxisanteil und einer Vielzahl an Übungen am Simulator, aber auch im Wet Lab sowie einer Assistenz während einer robotischen Operation [13]. Moglia et al. konzipierten demgegenüber ein strukturiertes 4‑stündiges Training sowohl am Laparoskopie- als auch am Robotiksimulator [12]. Auch hier geht den praktischen Anteilen ein theoretischer Abschnitt mit Prüfung voraus. Kuhn et al. legen in ihrem deutlich weiter gefassten Lehrkonzept zur „Medizin im digitalen Zeitalter“ nur in einem von fünf Modulen den Fokus auf die Robotik, neben Virtual und Augmented Reality [9]. Es sollen nicht nur Basisfertigkeiten am Simulator für robotische Chirurgie geübt werden, sondern auch immersive Simulationen laparoskopischer Cholezystektomien oder eine leberchirurgische OP-Planung mittels Augmented Reality durchgeführt werden. Auch wenn sich diese Programme teils deutlich von dem hier präsentierten Wahlfach unterscheiden, enthalten sie sowohl theoretische als auch möglichst viele praktische Anteile. Mullens et al. bieten ebenso eine Hospitation während einer robotischen Operation an.

In der theoretischen Eingangsprüfung unseres Wahlfachs (s. Supplement) konnten fast alle Teilnehmende ohne Vorbereitung bereits mehr als die Hälfte der Fragen beantworten, was auf ein gewisses Maß an Vorwissen hindeutet. Im Ausgangstestat wurden jedoch im Median 3,5 von 10 Fragen mehr richtig beantwortet. Folglich fand eine nachhaltige Vermittlung theoretischen Wissens statt, da zu diesem Zeitpunkt der Vortrag über die theoretischen Grundlagen bereits 4 Wochen zurücklag. An dieser Stelle sei explizit hervorgehoben, dass im Theorieteil nicht nur die Vor- sondern auch Nachteile roboterassistierten Operierens (insbesondere die im Vergleich zur Laparoskopie höheren Kosten) explizit diskutiert wurden, um den Studierenden ehrliche und vorurteilsfreie Einblicke zu geben.

Bei den praktischen Übungen am robotischen Simulationssystem direkt an der Operationskonsole war nur in 2 von 3 Fällen eine signifikante Verbesserung der Gesamtpunktzahl bei den Teilnehmenden messbar. Die Auswahl der Übungen zielte darauf ab, grundlegende Fähigkeiten im Umgang mit der robotischen Operationskonsole zu testen (Clutch: Verwendung der Kupplung, Camera 0: Steuerung der Kamera, Sea Spikes 1: Bewegung der Greifarme). Die Übung „Clutch“ ist aber offensichtlich so wenig anspruchsvoll, dass eine tatsächliche Verbesserung in der Bedienung anhand der Gesamtpunktzahl nicht abzubilden ist: Auch ohne vorherige Einweisung wurden bei der allerersten Durchführung im Median 98 von 100 möglichen Punkten erreicht. Die mit den Instrumenten zurückgelegte Bewegungsstrecke während der Übung verdeutlicht jedoch, dass sich die Bewegungsökonomie der Teilnehmenden verbesserte, da die zurückgelegte Strecke um 30 % abnahm. Dass sich die Teilnehmenden durch das Training in ihren praktischen Fähigkeiten verbesserten, ist nicht verwunderlich, war zu erwarten und konnte bereits bei anderen laparoskopischen Simulatortrainings gezeigt werden [6, 14]. Dabei muss hervorgehoben werden, dass es letztlich unerheblich zu sein scheint, ob komplett virtuell am Simulator oder im Wet Lab bspw. an echtem biologischen Gewebe geübt wird, denn der Lernzuwachs ist vergleichbar [1]. In zukünftigen Wahlfächern könnte insofern also nicht nur rein virtuell, sondern auch mit den eigens dafür konzipierten Übungsinstrumenten für das robotische Operationssystem an einem Hands-on-Phantommodell geübt werden.

Als organisatorischer Flaschenhals im Praxisteil stellte sich der Zugang zum Simulatorsystem dar, weil zumindest in unserer Klinik die Robotersysteme regelhaft im Tagesprogramm ausgelastet sind. Der DaVinci® SimNow®-Simulator als Nachfolger des DaVinci® Skills®-Simulator wird ebenfalls auf der Rückseite der Operationskonsole befestigt und ermöglicht das Erlernen grundlegender Steuerungsfunktionen der Konsole bis hin zur Simulation komplexer Operationen (bspw. der radikalen Prostatektomie). Er kann allerdings nur dann verwendet werden, wenn die Konsole nicht gerade bei einer Operation eingesetzt wird. Daher wurde der Beginn des Wahlfachs auf den späten Nachmittag angesetzt und damit einen Zeitpunkt, zu dem das reguläre Operationsprogramm bereits beendet ist. Im Optimalfall kann natürlich auf ein System zurückgegriffen werden, das ausschließlich dem Trainieren dient, was aber im Falle des hier eingesetzten robotischen Simulationssystems mit hohen fünfstelligen Kosten verbunden ist, da eine eigene Operationskonsole benötigt wird. Als Alternativen existieren auch Standalone-Systeme von anderen Anbietern, wie der dV-Trainer® (Mimic Technologies, Seattle, WA, USA) oder RobotiX Mentor® (3D Systems, ehemals Simbionix, Beit Golan, Israel; [10, 11, 20]). Diese Systeme ermöglichen ebenfalls ein Erlernen der grundlegenden Funktionen der Robotersysteme mit entsprechenden Trainingsprogrammen, wobei diese durchaus unterschiedlich strukturiert sind und ebenfalls schnell mehr als 100.000 US$ kosten [2]. Da immer nur ein Teilnehmender alleine an der Konsole üben kann, haben wir die Gruppe von 10 Studierenden auf zwei Teams à 5 Personen an den Praxisterminen verkleinert, um die Wartezeiten zu reduzieren. Zusätzlich wurde die Operationskonsole an einen externen Monitor angeschlossen, damit die Wartenden dem Übenden zuschauen können und daraus einen zusätzlichen Lerneffekt erzielen. Es ist natürlich auch denkbar, mehrere Übungsstationen gleichzeitig anzubieten, zwischen denen rotiert wird (bspw. unter Einbeziehung studentischer Hilfskräfte). Diese studentischen Hilfskräfte könnten dann auch das ärztliche Personal bei der Durchführung des Wahlfachs entlasten, zumal sich der Zeitaufwand in der Vor- und Nachbereitung als nicht unerheblich erwies. Darüber hinaus könnte das Wahlfach gleichzeitig als Rekrutierungsweg für zukünftige Doktorandinnen und Doktoranden dienen, indem die Teilnehmer für eine Promotionsstelle angeworben werden, im Folgenden selber ein Wahlfach leiten und später andere Doktorandinnen und Doktoranden im Rahmen eines Mentoringprogramms betreuen. Schließlich erscheint aus der wissenschaftlichen Perspektive heraus ein solches Lehrkonzept auch gut für die Durchführung von Trainingsstudien an Robotik- oder Laparoskopietrainern geeignet, die aufgrund ihres zeitlichen Aufwands kaum alleine von Ärzten geleitet werden können [15, 17].

Das Wahlfach sollte aber nicht nur theoretische und praktische Anteile enthalten, sondern auch eine Hospitation während einer robotischen Operation. Diese stellt eine sinnvolle Ergänzung dar, weil gewonnenes Wissen und erlernte Fertigkeiten direkt auf die intraoperative Situation übertragen werden können, was sehr positiv evaluiert wurde. Zum gegenwärtigen Zeitpunkt haben noch nicht alle Teilnehmenden während einer robotischen Operation hospitiert. Dies ist damit zu erklären, dass das Wahlfach nicht wie geplant zu Semesteranfang starten konnte und sich in den vergangenen Wochen fast alle Teilnehmenden auf Prüfungen am Semesterende vorbereiten mussten.

Einen für Urologen/innen berufspolitisch äußerst bedeutsamen Aspekt ist der Umstand, dass die Bereitschaft, möglicherweise in der Urologie tätig zu werden, am Ende des Wahlfachs bei den Teilnehmenden signifikant höher ausfiel. Die Arbeitsgemeinschaft Junge Urologen hat im Rahmen der „Zukunftsoffensive 2025“ klar zur Thematik der Nachwuchsförderung Stellung genommen und eine verstärkte Studentenbindung unter dem Slogan „Urologen der Zukunft“ als eines der Hauptziele benannt [19]. Die Faszination an der robotischen Chirurgie stellt eine bedeutsame Facette unseres Fachbereichs dar, die zukünftig noch stärker dafür eingesetzt werden sollte, aktiv für und um den urologischen Nachwuchs zu werben. In diesem Zusammenhang ist es sicher kein Zufall, dass das auf dem 71. Jahreskongress der DGU in Hamburg im Jahr 2019 vorgestellte Image-Video für unseren Fachbereich mit dem Motto „Die Vielfalt wartet. Worauf wartest du?“ als Teil der „Zukunftsoffensive Urologie“ direkt mit einer robotischen Operation beginnt. Insgesamt stieß unser strukturiertes Lehrkonzept mit mehr als 30 Anfragen für 10 Plätze auf großes Interesse. Es besteht offensichtlich ein großer Bedarf von studentischer Seite, sich in der robotischen Chirurgie zu bilden, weswegen bisher eine eigene Vorlesung über urologische Robotik gehalten wurde. Auch andere Fachbereiche haben das Potenzial der Faszination um robotische Chirurgie bereits erkannt und möchten dieses zukünftig stärker nutzen [8].

Als Limitation dieser Arbeit ist zu benennen, dass es sich hierbei um die allererste Durchführung mit einer limitierten Zahl an Teilnehmenden handelt, die Ergebnisse sind somit als präliminär zu bewerten. Es besteht die Möglichkeit, dass ein Selektionsbias in der Gruppenzusammensetzung vorliegt, weil bei den Teilnehmenden möglicherweise eine ohnehin schon vergleichsweise hohe Affinität zu chirurgischen Fächern bestand und deswegen die Effekte des Trainings überschätzt werden. Hierbei ist einschränkend anzumerken, dass vor dem Wahlfach zumindest bei den meisten Teilnehmern keine besondere Verbindung zur Urologie bestand. Eine unmittelbare Übertragbarkeit auf andere Kliniken ist aufgrund der hohen Kosten für ein robotisches Simulatorsystem nur bedingt gegeben, sofern dieses noch angeschafft werden müsste. Alternativ bieten sich hier natürlich auch Laparoskopietrainer an, die mit weniger Kosten verbunden sind – aber das robotische Add-on nicht bieten können. Nicht zuletzt wird sich erst in der Zukunft zeigen, ob die Teilnehmenden tatsächlich eine Famulatur oder ein Teil des praktischen Jahres in der Urologie ableisten – oder gar in einer urologischen Klinik anfangen werden, zu arbeiten. Dieser longitudinale Aspekt lässt sich nur im Verlauf beantworten und steht noch aus.

## Ausblick

Diese ersten Erfahrungen mit dem Wahlfach „Robotische Chirurgie“ zeigen, dass offensichtlich ein Bedarf von studentischer Seite besteht, sich in der roboterassistierten Chirurgie zu bilden. Das Wahlfach ermöglicht einerseits, den Studierenden theoretische wie praktische Inhalte der Robotik zu vermitteln. Andererseits erscheint es auch als ein gutes berufspolitisches Instrument, auf unser Fachgebiet aufmerksam zu machen, Interesse zu wecken und die Faszination an der robotischen urologischen Chirurgie zu transportieren. Darüber hinaus erscheinen Wahlfächer wie diese als geeignetes Medium, wissenschaftliche Fragestellungen bspw. zu Lernkurvenanalysen zu beantworten. Aus diesem Grund wird es zumindest in unserer Klinik kein einmaliges Angebot bleiben, und zukünftige Wahlfächer „Robotische Chirurgie“ werden folgen.

### Infobox 1 Mehr Informationen zum Thema

- DRUS robotisches Curriculum: https://www.dgru.de/
- ERUS robotisches Curriculum: http://uroweb.org/section/erus/education
- DGU-Imagefilm „Die Vielfalt wartet – worauf wartest du?“: https://www.youtube.com/watch?v=omEGZcgU754 (Alternativ unter urologenportal.de > Fachbesucher > DGU-Imagefilm)

## Fazit für die Praxis

- Vor dem Hintergrund der weiten Verbreitung roboterassistierter Operationen ist die robotische Chirurgie im Lehrplan heutiger Medizinstudierender unterrepräsentiert.
- Es besteht offensichtlich ein Bedarf von studentischer Seite, sich entsprechend zu bilden.
- Ein Wahlfach „Robotische Chirurgie“ erscheint in diesem Zusammenhang als geeignetes Format, theoretische Grundlagen und praktische Fertigkeiten in der robotischen (urologischen) Chirurgie zu vermitteln.
- Organisatorisch bietet sich die Aufteilung in einen Theorie-, mehrere Praxisteile und eine Hospitation an.
- Die Gruppen sollten nicht zu groß sein, insbesondere im Praxisteil maximal 5 Personen umfassen.
- Jenseits der edukativen Perspektive kann ein solches Wahlfach auf die faszinierende Vielfältigkeit der Urologie aufmerksam machen und möglicherweise auch neue Kolleginnen und Kollegen gewinnen.

## Supplementary Information





## Abkürzungen

DGU: Deutsche Gesellschaft für Urologie
DGRU: Deutsche Gesellschaft für robotische Urologie
EAU: European Association of Urology
ERUS: European Association of Urology Robotic Urology Section
NKLM: Nationaler kompetenzbasierter Lernzielkatalog
OSCE: „Objective structured clinical examination“
PJ: Praktisches Jahr
