# Supplementary material for: Robot-assisted surgery as an elective—fascinating lesson(s)?
Source: Urologe A. 2022 Jan 17;61(4):400–6. [Article in German] doi: 10.1007/s00120-021-01756-6 (PMC9005389; doi:10.1007/s00120-021-01756-6)
Supplement: Supplementary file 1 [file 120_2021_1756_MOESM1_ESM.docx]

**Theoretische Prüfung an 🞎 Tag 1 | 🞎 Tag 6**

Name: __________________________

Matrikelnummer: __________________________

**1. Welche Aussage trifft nicht zu? Vorteil der DaVinci Operationstechnik ist/ sind:**

(A) 10-fache Vergrößerung gegenüber der offenen Operation

(B) Instrumente mit 7 Freiheitsgraden („EndoWrist system“)

(C) zitterfreies Operieren

(D) ein Operieren ohne Komplikationen

(E) ein Zugang über kleine Operationsschnitte (minimal-invasiv)

**2. Mithilfe welcher Zugangswege können Roboter-assistierte Eingriffe durchgeführt werden? (Mehrfachnennung möglich)**

(A) transperitoneal

(B) retroperitoneal

(C) transperineal

(D) transthorakal

(E) transoral

**3. Bei dieser klinischen Fachrichtung werden Operationsroboter vom Typ DaVinci nicht routinemäßig eingesetzt:**

(A) Urologie

(B) Allgemein- und Viszeralchirurgie

(C) Herz-Thorax-Chirurgie

(D) Hals-Nasen-Ohrenheilkunde

(E) Unfallchirurgie

**4. Welche Aussage zum Vergleich offener, laparoskopischer und Roboter-assistierter Chirurgie trifft nicht zu?**

(A) Als Nachteil robotischen Operierens gelten höhere Kosten.

(B) Ein Vorteil minimal-invasiver Operationsverfahren sind verminderte postoperative Schmerzen und eine schnellere Rekonvaleszenz.

(C) Als Nachteil offenen Operierens wird heutzutage eine oft unergonomische Arbeitsposition angesehen.

(D) Die Lernkurve laparoskopischen Operierens ist steiler als in der Robotik.

(E) Eingriffe können Roboter-assistiert im Vergleich zur Laparoskopie mit gleichwertigen Ergebnissen durchgeführt werden.

**5. Welche dieser Kennzahlen zu DaVinci Operationssystemen trifft nicht zu?**

(A) Weltweit wurden bereits mehr als 5 bis 10 Millionen Patienten Roboter-assistiert operiert.

(B) Im Jahr 2000 wurde in Europa die 4. Generation von DaVinci Operationsrobotern freigegeben.

(C) Hochgerechnet wird alle 30 Sekunden eine Roboter-assistierte Operation begonnen.

(D) Der Marktwert von Intuitive Surgical beträgt etwa 75-80 Mrd. USD.

(E) Die neueste Generation von DaVinci Operationssystemen hat 4 Arme.

**6. Welcher der genannten robotischen urologischen Operationen hat den höchsten Schwierigkeitsgrad?**

(A) radikale Prostatektomie

(B) Pyeloplastik

(C) Nierenteilresektion

(D) radikale Zystektomie mit intrakorporaler Neoblase

(E) Nephrektomie

**7. Welche Aussage zur Geschichte der Roboter-assistierten Chirurgie trifft nicht zu?**

(A) Das erste DaVinci® Operationssystem von Intuitive Surgical wurde im Jahr 2000 von der FDA zugelassen.

(B) Das Wort “Roboter”, abgeleitet von tschechisch „robota“, sprich Arbeiter, wurde von Karel Capek im Jahr 1920 durch sein Drama „R.U.R. – Rossum’s Universal Robots“ geprägt.

(C) Einer der allerersten chirurgischen Operationsroboter war „Arthrobot“, ein kanadisches System zur Assistenz bei orthopädischen Interventionen im Jahr 1983.

(D) „PROBOT“ war der erste Operationsroboter in der Urologie und diente dazu, ab 1988 transurethrale Prostataresektionen durchzuführen.

(E) Direkt nach der Marktfreigabe des ersten DaVinci Operationssystems verklagte Intuitive Surgical den Mitbewerber Computer Motion, um seine Vorreiterstellung auszuweiten.

**8. Welcher der folgenden Aspekte steht nicht bei der Entwicklung neuer robotischer Operationssysteme im Fokus?**

(A) Eye-Tracking

(B) geringere Kosten

(C) Miniaturisierung

(D) größere Anzahl an Roboterarmen

(E) haptisches Feedback

**9. Welche dieser Funktionen steht dem robotische Operateur nicht an seiner Operationskonsole zur Verfügung?**

(A) Clutch: Reposition der Bedieninstrumente der Hände, ohne dass sich die Instrumente im Situs bewegen

(B) Kamera: Bewegung der Kamera, inkl. Rotation der Optik um 180°

(C) Autopilot: das System operiert selbstständig, muss aber dauerhaft vom Operateur überwacht werden

(D) Retraktionsarm: Wechsel zwischen den Operationsarmen

(E) Energiepedale: Applikation von monopolarem / bipolaren Strom

**10. Welche Aussage zum Port Placement und Docking bei Roboter-assistierten Operationen trifft nicht zu?**

(A) Ein Interkalieren der Instrumente während der Operation ist bei abdominellen Eingriffen durch Trokareinlage auf einer Linie vermeidbar.

(B) Spezielle Systeme ermöglichen auch das Operieren durch einen einzigen Port hindurch (LESS: laparoendoscopic single site surgery).

(C) Die Instrumente können heute beliebig zwischen den Armen vertauscht werden.

(D) Nach dem Andocken des Operationsroboters ist bei allen Systemen eine Änderung der Patientenlagerung problemlos möglich.

(E) Die Positionierung des Roboters kann mit einem Laser optimiert werden.

Punktzahl: ___ / 10 Punkten

**Lösungen:**

| 1: D  2: A-E alle richtig  3: E  4: D  5: B  6: D | 7: E  8: D  9: C  10: D |
| --- | --- |
